# Clinical and genetic analysis of a case of Gitelman syndrome accompanied with Graves disease and adrenocortical adenoma: A case report

**DOI:** 10.1097/MD.0000000000037770

**Published:** 2024-04-12

**Authors:** Yan Qiao, Jinghong Zhao, Ji Wu, Lewei Cao, Guiqin Song, Jingxin Mao

**Affiliations:** a Department of Endocrinology, Nanchong Central Hospital, The Second Clinical College, North Sichuan Medical College, Nanchong, China; b Department of Urology, Nanchong Central Hospital, The Second Clinical College, North Sichuan Medical College, Nanchong, China; c Institute of Basic Medicine and Forensic Medicine, North Sichuan Medical College, Nanchong, China; d Chongqing Medical and Pharmaceutical College, Chongqing, China; e College of Pharmaceutical Sciences, Southwest University, Chongqing, China.

**Keywords:** adrenal cortical adenoma, case report, cushing syndrome, Gitelman syndrome, Graves disease, SLC12A3 gene

## Abstract

**Rationale:**

Gitelman syndrome (GS), also known as familial hypokalemia and hypomagnesemia, is a rare autosomal recessive inherited disease caused by primary renal desalinization caused by impaired reabsorption of sodium and chloride ions in the distal renal tubules. We report a case of clinical and genetic characteristics of GS accompanied with Graves disease and adrenocorticotrophic hormone (ACTH)-independent adrenocortical adenoma.

**Patient concerns:**

The patient is a 45 year old female, was admitted to our hospital, due to a left adrenal gland occupying lesion as the chief complaint.

**Diagnosis:**

The patient was finally diagnosed as GS with Graves disease and adrenocortical adenoma.

**Interventions:**

Potassium magnesium aspartate (1788 mg/d, taken orally 3 times a day (supplement a few times a day, intake method, treatment duration). Contains 217.2 mg of potassium and 70.8 mg of magnesium, and potassium chloride (4.5 g/d, taken orally 3 times a day (supplement a few times a day, intake method, and treatment duration); Potassium 2356 mg), spironolactone (20 mg/d, taken orally once a day (supplement a few times a day, intake method, treatment duration). After 3 months of treatment, the patient’s blood potassium fluctuated between 3.3–3.6 mmol/L, and blood magnesium fluctuated between 0.5–0.7 mmol/L, indicating a relief of fatigue symptoms.

**Outcomes:**

On the day 6 of hospitalization, the symptoms of dizziness, limb fatigue, fatigue and pain were completely relieved on patient. In the follow-up of the following year, no recurrence of the condition was found

**Lessons:**

The novel c.1444-10(IVS11)G > A variation may be a splicing mutation. The compound heterozygous mutations of the SLC12A3 gene may be the pathogenic cause of this GS pedigree.

## 1. Introduction

Gitelman syndrome (GS) (OMIM: 263800) is an autosomal recessive disorder caused by structural and/or functional abnormalities in the thiazide diuretic sensitive sodium chloride co-transporter encoded by the SLC12A3 gene mutation, characterized by hypokalemic alkalosis, hypomagnesemia, and low urinary calcium.^[1,2]^ There is significant heterogeneity in the clinical characteristics of GS patients.^[3]^ There have been no reports of GS combined with Graves disease and adrenocortical adenoma both domestically and internationally. This study aims to analyze the clinical characteristics of an extremely rare patient with Graves disease and adrenocortical adenoma with GS, and perform whole exome sequencing to screen for variant genes related to its pathogenesis, providing a basis for GS screening and genetic counseling. This paper reports a case of GS accompanied with Graves disease and adrenocorticotrophic hormone (ACTH)-independent adrenocortical adenoma.

## 2. Case presentation

The patient is a 45 year old female, was admitted to our Department of Endocrinology of hospital on December 21, 2020, due to a left adrenal gland occupying lesion as the chief complaint. Patient has obvious symptoms such as dizziness, limb fatigue, fatigue and pain. Starting from 1 year ago, there were obvious symptoms such as dizziness, limb fatigue, fatigue, and no symptoms such as palpitations, shortness of breath, thirst, numbness of hands and feet. Patient with 1 year history of hypertension, no history of diuretic use. Patients having 1 son and 1 daughter, she ceased menstruation at the age of 38. The patient’s sister has a history of hypokalemia, and there are no abnormalities in thyroid and adrenal function.

Blood pressure was 141/90 mm Hg, body mass index was 29.5 kg/m^2^, centripetal obesity, full moon face, buffalo back, thin skin, no purple lines. No protruding eyes, small whiskers visible on the lips. Thyroid gland with degree I enlargement, soft texture, no tenderness, heart rate of 102 beats/min, regular rhythm, and normal limb muscle strength.

### 2.1. Blood analysis result

Blood potassium 2.22 (normal reference values 3.5–5.3, the same below) mmol/L, blood magnesium 0.49 (0.75–1.02) mmol/L, blood chlorine 91.9 (99–110) mmol/L, fasting blood glucose 6.5 (3.9–6.1) mmol/L, postprandial blood glucose 10.4 (<7.8) mmol/L, 24-hour urine potassium 92 (25–125) mmol, 24-hour urine chlorine 409 (110–250) mmol, 24-hour urine calcium 0.3 (2.5–7.5) mmol. pH 7.51 (7.35–7.45), standard bicarbonate 31.1 (21–25) mmol/L.

### 2.2. Thyroid function analysis result

TSH < 0.005 (0.27–4.20) mIU/L, FT3 9.59 (3.1 6.8) pmol/L, FT4 47.6 (12.0 22.0) pmol/L, thyroid peroxidase antibody 11.3 (0–34) IU/mL, thyrotropin receptor antibody 7.98 (0–1.75) IU/L.

### 2.3. Other inspections and analyses result

Renin > 500 (4–24) pg/mL, aldosterone (standing) 227.4 (10–160) pg/mL. The diurnal rhythm of cortisol disappears, and ACTH is inhibited: serum cortisol at 8:00, 16:00, and 24:00 is 18.71, 18.12, and 17.17 µg/dL, respectively; ACTH at 8:00, 16:00, and 24:00 is 2.40 pg/mL, respectively. 1.07, 1.16 pg/mL. The low-dose overnight dexamethasone inhibition test was not inhibited: serum cortisol (after inhibition) 18.89 µg/dL. The high-dose (16 mg) dexamethasone inhibition test was not inhibited: serum cortisol (after inhibition) was 17.62 µg/dL. The levels of free methoxy norepinephrine and free methoxy epinephrine in the blood are normal.

**Figure 1.**
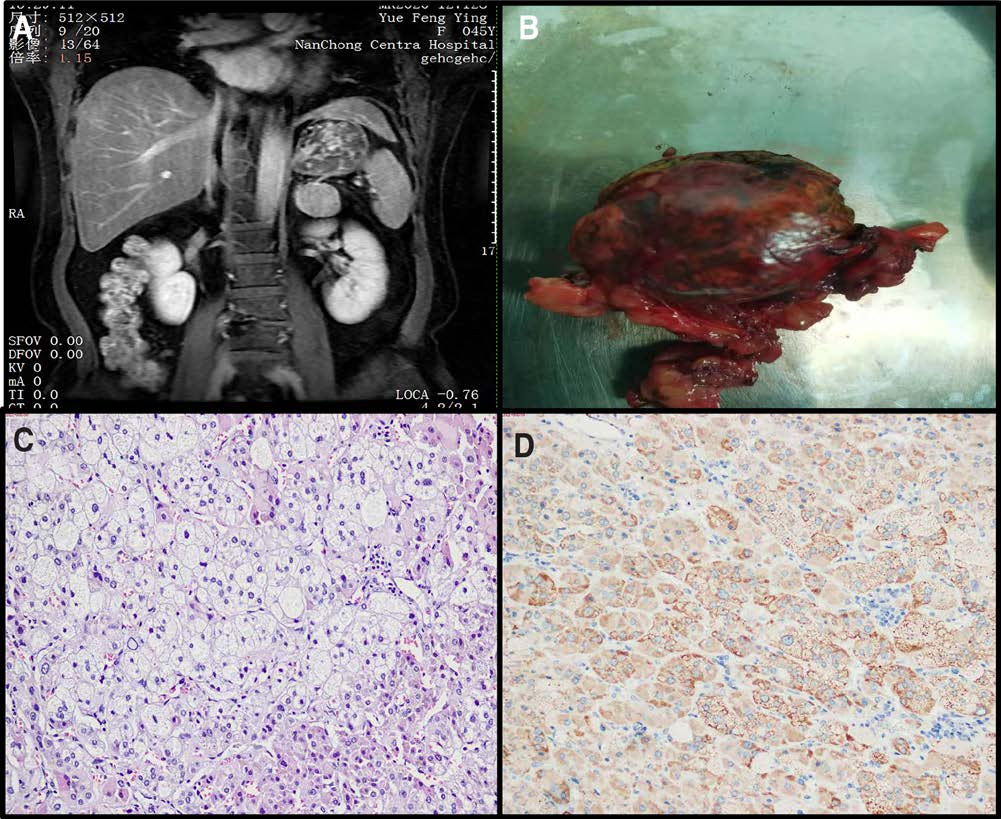
Patient related auxiliary examination result (A) Tumor MRI imaging, (B) Tumor gross specimen, (C) Tumor HE staining × 100, (D) Tumor immunophenotype × 100.

Adrenal Magnetic resonance imaging result showed that left lateral adrenal branch 3.8 cm × 3.2 cm space occupying (Fig. 1A). After 131I treatment, the patient’s thyroid function was basically normal, and laparoscopic left adrenal mass resection was performed. Pathological examination showed a grayish brown nodule with a size of 4.5 cm × 3.5 cm × 2.0 cm, weighing 35 g, with a gray brown gray yellow cut surface, solid and soft texture (Fig. 1B). After HE staining, pathological findings under the microscope indicate that is adrenocortical adenoma (Fig. 1C). Immunohistochemical staining results revealed that mean A (+), *α*-inhibin (+), CR (+), *β*-catenin (cytoplasm+), CgA (-), Ki-67 (+, <5%) (Fig. 1D).

2 mL of peripheral venous blood samples were collected from the patient, their parents, and their sister, anticoagulate with EDTA, and extract Deoxyribo nucleic acid. Using the IDT xGen exome research panel v2.0 full exon capture chip, the proband was subjected to deep whole exome sequencing and screening. After analyzing the data, the screened candidate variants were subjected to Sanger sequencing using the ABI 3730 sequencer (American company ABI, Beijing Full Spectrum Medical Laboratory, Building E2, Biopharmaceutical Science and Technology Innovation Park, No. 88, 6th Street, Yizhuang, Beijing, China). According to the American College of Medical Genetics and Genomics (ACMG) guidelines for interpreting the pathogenicity of gene mutations, the mutations are classified to provide evidence for diagnosis.

**Figure 2.**
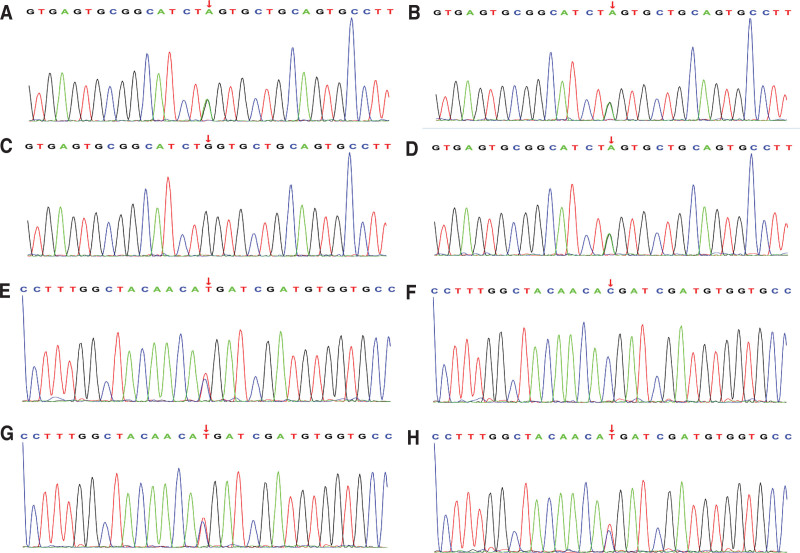
SLC12A3 gene sequencing map c.1444-10 (IVS11) G > A variation in (A) patients, (B) father, (C) mother, and (D) sister. SLC12A3 gene sequencing mapc.179(exon1) C > T/p.T60M variation in (E) patients, (F) father, (G) mother, and (H) sister.

The sequencing results showed that the patient and her sister had a compound heterozygous variation of c.1444-10 (IVS11) G > A, c.179 (exon1) C > T/p.T60M in the SLC12A3 gene (Fig. 2). Among them, the c.1444-10 (IVS11) G > A variant was inherited from the father (Fig. 2A–D), and the c.179 (exon1) C > T/p.T60M variant was inherited from the mother (Fig. 2E–H). The mutation c.1444-10 (IVS11) G > A located in the intron is not included in the common human carrier frequency database, such as the thousand human genome, gnomAD, etc. There are no relevant reports in the literature, and it is a new mutation. Another variant, c.179 (exon1) C > T/p.T60M, is a clearly reported GS pathogenic mutation and a compound heterozygous variant composed of c.1444-10 (IVS11) G > A, which has not been reported in the past. The phenotype and genotype of the proband and its family members are consistent with co segregation. According to the guidelines for interpreting the pathogenicity of ACMG gene variants, the c.1444-10 (IVS11) G > A variant is rated as an uncertain variant that may affect splicing; c. 179 (exon1) C > T mutation is rated as a possible pathogenic variant, and the next step is to consider conducting in *vivo* or in *vitro* functional tests to provide evidence for determining pathogenicity. The test results did not indicate the presence of genetic lesions in Graves disease, adrenocortical adenoma, or CLCNKB gene mutations. The results of whole exome sequencing showed that the SLC12A3 gene of the patient and her sister had a compound heterozygous variation of c.1444-10 (IVS11) G > A, and c.179 (exon1) C > T (p.T60M). Among them, the c.1444-10 (IVS11) G > A variant was inherited from the father, and the c.179 (exon1) C > T (p.T60M) variant was inherited from the mother. The variant of her sister is similar to her.

The patient with adrenal incidentaloma was diagnosed as Graves disease, ACTH-independent cushing syndrome, hypokalemia, and hypomagnesemia by clinical examination. Magnetic resonance imaging scan found that a 3.8 × 3.2 cm mass located in the left adrenal gland. The mass was surgically removed and adrenocortical adenoma was proved by pathologic microscopy. Deoxyribo nucleic acid sequencing revealed that patient and her sister have carried compound heterozygous mutations of the SLC12A3 gene, namely c.1444-10(IVS11)G > A and c.179(exon1)C > T/p.T60M, which were respectively inherited from their father and mother. This patient and her sister were diagnosed with GS. Her sister had hypokalemia and normal thyroid and adrenal functions.

Therapeutic intervention as following: potassium magnesium aspartate (1788 mg/d, taken orally 3 times a day (please supplement a few times a day, intake method, treatment duration). Contains 217.2 mg of potassium and 70.8 mg of magnesium, and potassium chloride (4.5 g/d, taken orally 3 times a day (please supplement a few times a day, intake method, and treatment duration); Potassium 2356 mg), spironolactone (20 mg/d, taken orally once a day (please supplement a few times a day, intake method, treatment duration). After 3 months of treatment, the patient’s blood potassium fluctuated between 3.3–3.6 mmol/L, and blood magnesium fluctuated between 0.5–0.7 mmol/L, indicating a relief of fatigue symptoms. On the day 6 of hospitalization, the symptoms of dizziness, limb fatigue, fatigue and pain were completely relieved on patient. This study was approved by the Ethics Committee of Nanchong Central Hospital (No. 2023007). In addition to approving study protocols, informed consent was obtained from all subjects involved in this study. Furthermore, we confirm that the data associated with this manuscript is anonymized.

## 3. Discussion

The SLC12A3 gene is located on chromosome 16q13 and contains 26 exons. At present, 593 mutation sites in the SLC12A3 gene have been identified, including missense mutation, nonsense mutation, splicing mutation, deletion mutation, frameshift mutation, and insertion mutation.^[4]^ The common type of variation is compound heterozygous variation.^[5–7]^ It was reported that GS is more common in Asia, with a prevalence rate of 10.3/10,000 in the Japanese population.^[8]^ The onset age and clinical manifestations of GS are highly heterogeneous, with typical manifestations including low blood potassium, low blood magnesium, low blood chloride, low urinary calcium, low blood volume, and activation of the renin angiotensin aldosterone system.

In the present study, patients with GS combined with Graves disease, and ACTH dependent adrenocortical adenoma, are very rare and have severe and complex clinical phenotypes, making diagnosis difficult. GS needs to be distinguished from thyroid toxic periodic paralysis caused by Graves disease, which mainly affects Asian males. Currently, there are almost no reports of female patients, mainly manifested as recurrent hypokalemia, thyroid toxicity, and muscle weakness, and such patients have low urinary potassium. Excessive thyroid hormones can lead to an increase in Na^+^-K^+^-ATPase activity, accelerate potassium ion excretion and intracellular transfer, leading to hypokalemia.^[9]^ GS combined with Graves disease is very rare in clinical practice.^[10,11]^ The patient in this study is a female, with hypokalemia and increased urinary potassium. After controlling thyroid function, there is still difficult to correct hypokalemia, indicating that the patient’s hypokalemia may have other causes. In addition, GS also needs to be distinguished from adrenal diseases such as adrenocortical adenoma. In this study, patients still have adrenocortical adenoma that mainly secrete cortisol, causing adrenocortical adenoma, symptoms such as muscle weakness, hypokalemia, hypertension, impaired glucose regulation, and menopause. Due to the contradiction between hypertension and the decrease in blood pressure caused by GS tubular desalinization, the condition is mixed, increasing the difficulty of diagnosis. Patients with cushing syndrome have 11-b hydroxysteroid dehydrogenase deficiency, which reduces the amount of cortisol involved in metabolism and conversion into cortisone, and increases the amount of combined with mineralocorticoid receptor, thus enhancing the effect of mineralocorticoid like on the epithelial cells of distal convoluted tubules and collecting ducts, causing increased potassium excretion and sodium retention, leading to hypokalemia and hypertension.^[12]^ The merger of GS with non ACTH dependent CS and adrenal cortisol secreting adenoma has not been reported in the past. In this study, patients were found to have low blood magnesium, low blood chlorine, and low urinary calcium upon examination. It is necessary to be cautious of adrenocortical adenoma merging with GS. After adrenocortical adenoma symptoms such as adrenocortical adenoma resection and hypertension were controlled, severe hypokalemia still exists, further supporting adrenocortical adenoma merging with GS. There is currently no research indicating a common pathogenesis among GS, Graves disease, and non ACTH dependent adrenocortical adenoma. In addition, GS needs to be differentiated from Bartter syndrome, another type of low potassium desalinated renal tubular disease. The classic Bartter syndrome (Bartter syndrome type III) is caused by a mutation in the gene CLCNKB encoding the chloride ion channel ClC-Kb located in the thick segment of the ascending branch of the medullary loop.^[13]^ Patients with Bartter’s syndrome are more likely to experience growth and development delay before the age of 3, with normal blood magnesium levels and normal or high urinary calcium levels. Chloride ion clearance tests and genetic testing can further differentiate.

The “Consensus of Experts on the Diagnosis and Treatment of Gitelman Syndrome” in China recommends that when patients have characteristic clinical manifestations and laboratory tests, a family investigation should be conducted, and genetic testing is recommended to obtain a diagnosis.^[14]^ The patient had hypokalemic alkalosis, low blood magnesium, high renin, low blood chlorine, low urinary calcium, and increased urinary potassium excretion, and their sister also had hypokalemia, which supports the diagnosis of GS and was confirmed through genetic testing in the present study. It is speculated that the clinical symptoms of the patient and her sister are related to the complex heterozygous variation of the SLC12A3 gene c.1444-10 (IVS11) G > A, c.179 (exon1) C > T (p.T60M) shown in sequencing results.

Previous research has shown that p.T60M is a common type of amino acid mutation in Chinese people, which is a phosphorylation site at the amino end of the protein encoded by the SLC12A3 gene. The p.T60M mutation can affect the stability and transport activity of thiazide diuretic sensitive sodium chloride co-transporter.^[15]^ In recent years, studies have also reported that intron variation can cause new splicing sites, leading to abnormal splicing of the SLC12A3 gene exon.^[16,17]^ The patient, sister, and father in this study all detected the c.1444-10 (IVS11) G > A variant, which has not been reported yet. It was predicted by splicing site hazard prediction software (MaxEntScan, GTAG, etc.) to be a harmful variant that may affect splicing, and further functional validation is needed. According to ACMG related guidelines, it is rated as a mutation with unknown significance. Therefore, this study speculates that the compound heterozygous variation of c.1444-10 (IVS11) G > A and c.179 (exon1) C > T (p.T60M) in the patient’s SLC12A3 gene is the genetic cause of GS, further expanding the SLC12A3 gene variation spectrum and providing more information for GS screening and genetic counseling.

## 4. Conclusions

This rare case report of GS combined with Graves disease and adrenocortical adenoma has not been reported domestically or internationally. The c.1444-10 (IVS11) G > A mutation in the SLC12A3 gene is a new variant, possibly a splicing variant, and a compound heterozygous variation with c.179 (exon1) C > T/p.T60M is a suspected pathogenic variant in the GS family.

## Author contributions

**Conceptualization:** Yan Qiao, Jingxin Mao.

**Data curation:** Ji Wu, Lewei Cao.

**Formal analysis:** Ji Wu.

**Methodology:** Yan Qiao, Jingxin Mao.

**Project administration:** Yan Qiao, Guiqin Song.

**Software:** Jinghong Zhao, Lewei Cao.

**Supervision:** Lewei Cao.

**Visualization:** Jinghong Zhao.

**Writing – original draft:** Yan Qiao, Jingxin Mao.

**Writing – review & editing:** Jingxin Mao, Guiqin Song.
